# Genetic characterization of *Echinococcus* isolates from various intermediate hosts in the Qinghai-Tibetan Plateau Area, China

**DOI:** 10.1017/S0031182019000544

**Published:** 2019-06-21

**Authors:** Xiumin Han, Yingna Jian, Xueyong Zhang, Liqing Ma, Wenjun Zhu, Qigang Cai, Shile Wu, Xiangqian Wang, Bingqiang Shi

**Affiliations:** 1 Clinical Research Institute of Hydatid Disease, Qinghai Provincial People's Hospital, Xining 810007, China; 2 Qinghai Academy of Animal Sciences and Veterinary Medicine, Qinghai University, State Key Laboratory of Plateau Ecology and Agriculture, Qinghai University, Xining 810016, China

**Keywords:** *Cox*1 gene, genetic variation, genotype, haplotypes, hydatid cyst, Qinghai-Tibetan Plateau Area

## Abstract

This study examined *Echinococcus* spp. genotypes and genetic variants isolated from humans as well as domestic and wild animals from the Qinghai-Tibetan Plateau Area using the *cox*1 gene. All samples except the pika isolates were identified as the *Echinococcus granulosus* sensu stricto. Sixteen different haplotypes with considerable intraspecific variation were detected and characterized in mitochondrial *cox*1 sequences. The parsimonious network of *cox*1 haplotypes showed star-like features, and the neutrality indexes computed *via* Tajima's *D* and Fu's *F*s tests showed high negative values in *E. granulosus* s. s., indicating deviations from neutrality; the Fst values were low among the populations, implying that the populations were not genetically differentiated. The pika isolates were identified as *E. multilocularis* and *E. shiquicus*. Only one haplotype was recognized in the pika isolates. *E. granulosus* s. s. was the predominant species found in animals and humans, followed by *E. multilocularis* and *E. shiquicus*, with high genetic diversity circulating among the animals and humans in this area. Further studies are needed to cover many sample collection sites and larger numbers of pathogen isolates, which may reveal abundant strains and/or other haplotypes in the hydatid cysts infecting human and animal populations of the QTPA, China.

## Introduction

Echinococcosis is caused by the metacestodes of *Echinococcus* spp. and is parasitic in the livers, lungs and/or other organs of humans and animals, resulting in serious zoonotic parasitic disease. Echinococcosis epidemic diseases not only threaten and harm the health of humans and animals but also seriously hinder the production of animal husbandry and affect the development of the national economy (Budke *et al*., 2006; Qian *et al*., 2017). This disease is prevalent in Asia, South America, North Africa, Central Europe and other regions and causes harm throughout the world (Grosso *et al*., 2012). China, especially the Qinghai-Tibetan Plateau Area (QTPA), has one of the highest prevalence rates in the world (Wang *et al*., 2014). Echinococcosis identified as a zoonotic disease with a major impact on the public health of rural populations in Qinghai-Tibetan Plateau. As our previous study showed a high seropositive rate (37.0%) of echinococcosis in Qinghai-Tibetan primary school students (Han *et al*., 2018). Prevalence of *E. granulosus* in yaks, pigs and Tibetans investigated in Qinghai-Tibetan Plateau was 6.49, 7.27 and 1.83%, and Prevalence in yaks was 3.61, 9.66 and 6.33% in 2014, 2015 and 2016, respectively (Li *et al*., 2017). The action plan for prevention and treatment of echinococcosis was carried out by the People's Republic of China. A series of work was being implemented, such as the management and deworming of source of infection-dog, the vaccine immunization of the livestock, the implementation of livestock slaughter standard in slaughtering house (safety disposal of diseased organs), the management of patients (patient care and rescue), health education, people training and providing safe drinking water. The seven provinces and autonomous regions in northwestern China are epidemic areas of hydatid disease, endangering nearly 50 million people and 70 million livestock, which has resulted in direct economic losses of up to 30 billion Yuan (Qian *et al*., 2017).

*Echinococcus* spp. exhibit a fixed life cycle between definitive and intermediate hosts. Carnivores, such as dogs, foxes and wolves, are the definitive hosts of the parasites. The intermediate hosts involve different species and change in different environments (Romig *et al*., 2017). *Echinococcus* spp. can be infective in metacestode hosts, such as humans, livestock and some wild animals (Thompson, 2017). With the continuous improvement of molecular and genetic knowledge of parasites, identification and classification research has surpassed morphological study (Nakao *et al*., 2013). Genetic analysis employing mitochondrial genes (*cox*1, *nad*1, *cob* and *nad*5 gene) and ribosomal genes might reveal the biological relationships between *Echinococcus* spp. and strains (Marinova *et al*., 2017; Kinkar *et al*., 2018*a*). Mitochondrial DNA (mtDNA) is the most suitable genetic marker for the analysis of genetic diversity, genetic differentiation and evolution of species and is widely used in species classification (Umhang *et al*., 2014). There are five epidemic *Echinococcus* spp.: *E*. *granulosus* s. l., *E. multilocularis*, *E. oligarthrus*, *E. vogeli* and *E. shiquicus* (Nakao *et al*., 2013).

*E*. *granulosus* s. l. is a complex of different species including *E. granulosus* s. s.. *E*. *granulosus* s. s. includes the G1 and G3 genotype complex groups, with the G1 genotype (sheep strain) being prevalent worldwide (animals and humans) (Kinkar *et al*., 2018*b*). The G1 genotype is also the main epidemic strain of *E*. *granulosus* s. s. in China (Zhang *et al*., 2014). *E. granulosus* s. s. G1 infections of sheep, cattle and pigs have been reported in Argentina, Brazil, Chile and Mexico in the Americas as well, with high haplotype diversity (Laurimae *et al*., 2016). Similar results obtained from analyses of G1 infecting intermediate host species (cattle, sheep, human, wild boar, pig and goat) in Europe (Albania, Finland, Greece, Italy, Romania, Spain and Turkey) have revealed a complex phylogeography and high genetic variation (Kinkar *et al*., 2016). Previously, the G2 (Tasmanian sheep strain) is regarded as a separate genotype and has been found in France, Italy and Argentina (McManus and Thompson, 2003; Casulli *et al*., 2008); however, the genotype G2 is currecntly treated as an invalid genotype that is belonging to the genotype G3 cluster (Kinkar *et al*., 2017). The G3 genotype (buffalo strain) is prevalent in India and Iran (Grosso *et al*., 2012; Rostami *et al*., 2013). However, G3 is significantly less prevalent worldwide than G1, and also exhibits high genetic diversity (Kinkar *et al*., 2018*c*). Some recently obtained *E. granulosus* s. l. complex genotypes can be regarded as distinct species based on the differences in the morphology, host range and life cycles of these parasites, including *E. granulosus* s. s. (G1 and G3), *E. equinus* (G4), *E. ortleppi* (G5) (Thompson and McManus, 2002; Kinkar *et al*., 2017). However, the status of genotypes G6–G10 is still under dispute (Moks *et al*., 2008; Thompson, 2008; Saarma *et al*., 2009; Knapp *et al*., 2011, 2015; Lymbery *et al*., 2015). G6/G7 and G8/G10 can be regarded as two distinct species based on recent molecular evidence based on six nuclear genes; moreover, the marked biological differences exist between G6/G7 and G8/G10 (Laurimae *et al*., 2018). In addition, the genotype G9 has been characterised, but the status of G9 is still uncertain (Scott *et al*., 1997); analysis of phylogenetic systematics showed that the dubious G9 genotype can be included in G7 (Oksanen and Lavikainen, 2015), and maybe a variant of G7. The controversial genotypes remain to be discussed in future studies.

*Echinococcus* spp. isolates harbour a high degree of internal variation with substantial genetic differences and inter-isolate variation in development (such as the development of eggs) in different geographical environments and different hosts. These differences cause variation in propagation dynamics, pathogenicity, antigen-antibody reactions, clinical manifestations and chemotherapy responses between hosts (McManus and Thompson, 2003; Torgerson and Heath, 2003). There are a wide range of intermediate hosts showing adaptability to *E. granulosus* s. s. (Onac *et al*., 2013). In the long evolutionary process, variations also arise in the mutual adaptation of *Echinococcus* spp. Therefore, studies examining polymorphism in *Echinococcus* spp. might be directly related to the prevention of epidemics and treatment of local hydatid disease in the QTPA (Ma *et al*., 2015).

In this study, we analysed the genetic polymorphism of the mitochondrial *cox*1 gene of *Echinococcus* spp., combined with clinical data, to explore the intra-species variation of *Echinococcus* spp. and evaluate the population genetic structures of the three species in the QTPA. The resulting data and epidemiological information enabled us to propose evolutionary hypotheses regarding how three species of parasites have spread in the QTPA of China.

## Materials and methods

### Collection of samples

A total of 244 hydatid cysts were collected, including 93 human isolates (65 patients) obtained from surgical operations in the local hospitals of Qinghai Province and 38 sheep (28), 91 yak (64), and 22 wild pika (15) isolates obtained from the slaughterhouse between 2010 and 2017. The plateau pika was *Ochotona curzoniae*, which was identified according to the behavioural and morphological patterns of pikas by the local veterinarian. The yak species was *Bos grunniens* and the sheep were Tibetan sheep. Each single isolated cyst was regarded as an independent sample, and the contents of some hydatid cysts (except some lesions were calcification) were placed in tubes. In some cases, there was more than one lesion per animal/human, so two isolates were sampled from one animal/human. All the cyst samples were washed clean with phosphate-buffered saline (PBS) three times, and the samples were then preserved at −20 °C until being used for genomic DNA extraction.

### DNA extraction and PCR amplification

To extract genomic DNA from cysts, including the protoscoleces and germinal layers, each individual cyst was washed at least three times with sterile distilled water and centrifuged to remove the salt ions from PBS, after which genomic DNA was extracted according to the manufacturer's instructions (TIANamp Micro DNA Kit, Code: DP316, Tiangen, Beijing, China). The genomic DNA concentration was measured using a spectrophotometer (Merck Millipore, Frankfurter, Germany), and the DNA was then stored at −20 °C until being used for PCR amplification. A fragment of the mitochondrial genes was amplified from each sample using the primers described by Liu *et al*. and Bowles *et al*. for detection and analysis (Bowles *et al*., 1992, 1994; Liu *et al*., 2015). The PCR conditions and procedures were modified slightly, and the primers were *cox*1F: 5′- CCTGGATTTGGTATAATTAGTCA-3′ and *cox*1R: 5′- ATCATGCAAAAT/CATTATCT/CAACACACA-3′ (product = 366 bp). Each PCR mixture had a total volume of 50.0 *µ*L, containing 25.0 *µ*L of Premix Taq™ (TaKaRa Taq™ Version 2.0, Code: R004, Takara Bio Inc, Tokyo, Japan), 2.0 *µ*L of each primer (10.0 *µ*m), 2.0 *µ*L of template DNA, and 19.0 *µ*L of deionized distilled water. Positive and negative controls were run in parallel with the PCR amplification of the DNA samples. PCR amplification was carried out in a thermocycler (Mastercycler nexus GSX1, Eppendorf, Saxony, Germany) with a 5.0 min initial denaturation step at 95.0 °C; 35 cycles of a 35 s denaturation at 94.0 °C, a 45 s of annealing at 54.5 °C and a 40.0 s of extension at 72.0 °C; and a 10.0 min final extension at 72.0 °C. In the next step, the PCR products of the *cox*1 gene were electrophoresed in 1.2% agarose gels and stained with nucleic acid dyes. The PCR product bands were observed under UV light and recorded as digital images with a gel documentation system (BEIJING LIUYI BIOTECHNOLOGY CO., LTD., Beijing, China).

### Sequencing and alignment analysis

A total of 244 PCR products (product = 366 bp) from different hosts were sequenced using both the forward and reverse primers by the GENEWIZ Company (Suzhou, China). Sequences were identified and compared in the GenBank database through BLAST analysis (https://blast.ncbi.nlm.nih.gov/Blast.cgi?), developed by the National Center for Biotechnology Information (https://www.ncbi.nlm.nih.gov/). Amino acid sequences were inferred from the nucleotide sequences based on echinoderm and flatworm mitochondrial genetic codes using the ExPASy translate tool (https://web.expasy.org/translate/). Then, the sequences were subjected to multiple alignment by using the Clustal Omega alignment programme (http://www.ebi.ac.uk/Tools/msa/clustalo/) with reference sequences from different *E. granulosus* s. s. genotypes and *Echinococcus* spp..

### Data analysis

DnaSP 5.0 software was used to convert the fasta format (.fas) to the Network data format (.rdf) ARLEQUIN data format (.arp), and population diversity indexes (number of haplotypes and haplotype diversity) could also be calculated with this software. Then, we used NETWORK 5.0 (http://www.fluxus-engineering.com) to construct haplotype median-joining networks (Bandelt *et al*., 1999). Networks were constructed from the nucleotide sequences of the mitochondrial *cox*1 gene of *Echinococcus* spp. from all samples based on parameters of weights = 10 and epsilon = 0; nuclear data showed minimal variation and were not included. We computed population diversity indexes (number of haplotypes, haplotype and nucleotide diversity, and mean number of pairwise differences) within the different host groups identified from the phylogenetic analyses with the programme ARLEQUIN 3.5 (Excoffier and Lischer, 2010), which was also employed to calculate the neutrality indexes of Tajima's *D* (Tajima, 1989) and Fu's *F*s (Fu, 1997); finally, the degree of gene flow among the three host populations (human, yak and sheep) was estimated using a pairwise fixation index (Fst) as a relative measure of population differentiation, which was determined with the ARLEQUIN package.

## Results

### Sequencing analysis

PCR amplification of the *cox*1 gene was successfully performed for all of the isolated hydatid cysts. According to the *cox*1 gene nucleotide sequences obtained from the isolated samples, all the human isolates (*n* = 93), yak isolates (*n* = 91) and sheep isolates (*n* = 38) were identified as the *E. granulosus* s. s. G1 genotype. In addition, the pika isolates were identified as *E. multilocularis* (*n* = 16) and *E. shiquicus* (*n* = 6). *E. granulosus* s. s. G1 was clearly the most prevalent species in the animal and human isolates of hydatid cysts. None of the isolates from a given patient/animal occurred in a coinfection.

### Variation in nucleotide and amino acid sequences of *E. granulosus* s. s.

A 366-nucleotide consensus *cox*1 sequence was used to compare and to obtain the haplotypes (Farhadi *et al*., 2015). Based on the comparison data on *cox*1 gene sequences, 16 different haplotypes (G1s) were detected, which were designated as EgQH1 to EgQH16 [GenBank accession numbers MG674403-MG674418 (Table 1)]. A total of 34 point mutations were found within the haplotypes, consisting of 27 (79.4%) synonymous and 7 (20.6%) non-synonymous substitutions. For the non-synonymous mutations, the number of transition mutations was 5, and the number of transversions was 2; interestingly, the same transition mutation was found at position 56 of EgQH4 (C to T) and EgQH11 (C to T), and the same transition mutation was found at position 111 of ten haplotypes (T to C). Based on the sequence alignment, the EgQH7 haplotype was completely identical to the G1 (AF297617) reference genotype without base mutations; EgQH1, EgQH2, EgQH4, EgQH6, EgQH8, EgQH9, EgQH11, EgQH12, EgQH13, EgQH15 and EgQH16 showed very small nucleotide variations at either one or two positions, and seven haplotypes all included the non-synonymous substitution position 111 (T to C); another five haplotypes, EgQH3, EgQH5, EgQH7, EgQH10 and EgQH14, showed a large number of nucleotide variations (3 to 6) parallel to this, resulting in 1 to 2 different amino acid substitutions. Six nucleotide variations in EgQH10 led to only 2 amino acid substitutions, but two nucleotide variations in EgQH8 also generated 2 substitutions. There was only one transition mutation in EgQH4, EgQH5 and EgQH9, leading to the substitution of an amino acid. Among all 16 haplotypes, EgQH1 was the most common variant and was observed in 53 (21.7%) isolates, comprising 19 human, 21 yak and 13 sheep isolates. EgQH2 was the second most common variant, being found in 40 (16.4%) isolates: 14 human, 15 yak and 11 sheep isolates (Table 1). The next fourteen haplotypes (EgQH3–EgQH16) were found in 129 (52.9%) isolates. In addition, while haplotype EgQH1 was the most prevalent variant (22.5%) in the animal isolates, it was also observed (20.4%) in human isolates, and the second most frequent haplotype in the animal isolates was EgQH2.

**Table 1.** *Echinococcus* spp. haplotypes characterized by the partial mitochondrial cytochrome c oxidase subunit 1 (*cox*1) gene sequence (length = 366 bp) analysis

Haplotype of isolated Qinghai strains	Host origin (number)	Accession no.	Haplotype in the Fig. 1
EgQH1	Human (19), yak (21), sheep (13)	MG674403	EGH1
EgQH2	Human (14), yak (15), sheep (11)	MG674404	EGH3
EgQH3	Human (11), yak (13), sheep (5)	MG674405	EGH4
EgQH4	Human (9), yak (11), sheep (2)	MG674406	EGH7
EgQH5	Human (7), yak (9), sheep (3)	MG674407	EGH8
EgQH6	Human (6), yak (2), sheep (1)	MG674408	EGH9
EgQH7	Human (5), yak (4), sheep (0)	MG674409	EGH10
EgQH8	Human (3), yak (3), sheep (0)	MG674410	EGH11
EgQH9	Human (2), yak (1), sheep (1)	MG674411	EGH12
EgQH10	Human (3), yak (2), sheep (1)	MG674412	EGH15
EgQH11	Human (2), yak (2), sheep (0)	MG674413	EGH16
EgQH12	Human (2), yak (2), sheep (0)	MG674414	EGH17
EgQH13	Human (2), yak (2), sheep (0)	MG674415	EGH18
EgQH14	Human (3), yak (1), sheep (1)	MG674416	EGH19
EgQH15	Human (3), yak (1), sheep (0)	MG674417	EGH22
EgQH16	Human (2), yak (2), sheep (0)	MG674418	EGH23
EmQH1	Pika (16)	MG674419	EMH1
EsQH1	Pika (6)	MG674420	ESH1

### Haplotype networks

**Fig. 1.**
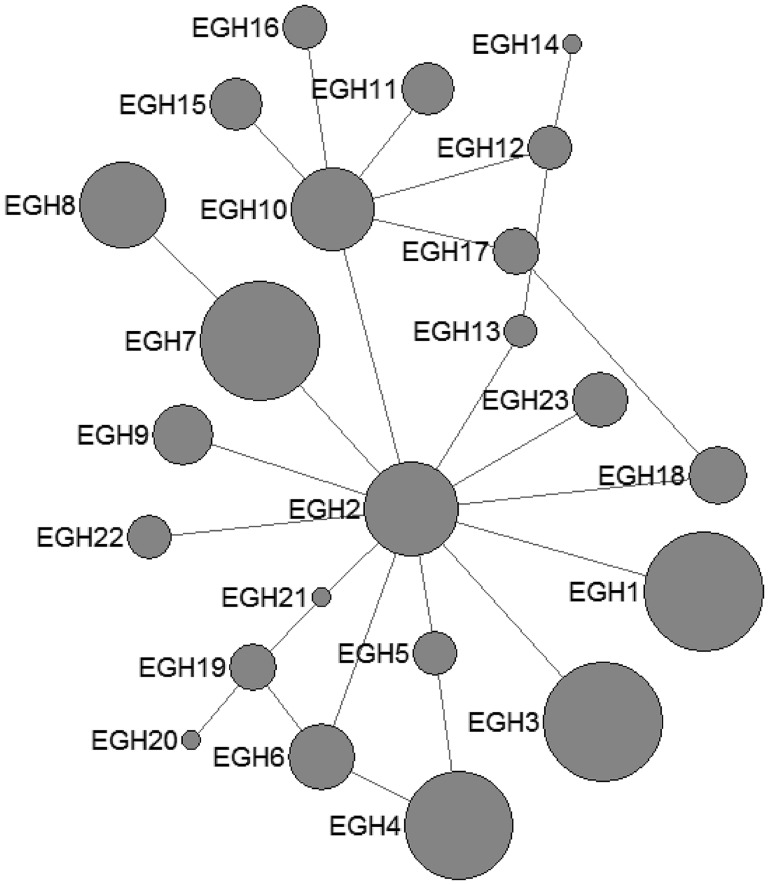
Haplotype network of the cytochrome c oxidase subunit 1 (*cox*1, 366 bp) gene of the *Echinococcus granulosus* sensu stricto complex from the QTPA of China (EGH1-EGH16) and other homologous sequences from GenBank constructed using NETWORK 5.0.

In *E. granulosus* s. s., 16 mtDNA haplotypes were found in 222 isolates from the QTPA of China. Nine haplotypes (EgQH1- EgQH 6, EgQH9, EgQH10 and EgQH14) were found in the populations of all three species (Fig. 1). Assuming that the ancestral haplotypes were still present in the recent populations, we constructed a statistical parsimony network to discriminate the genealogical relationships of the haplotypes among the hosts. The network showed a star-like configuration, with the common haplotype (G1) occupied the centre of the network, while G2 (EGH22) and G3 (EGH23) were linked to G1 *via* one mv1 (median vector) (Fig. 1). The population network presented a high-complexity structure, which included two main sub-groups (EGH4-EGH5 and EGH7-EGH12). However, the majority of haplotypes (EGH1-EGH16) and (EGH18-EGH21) all contained Chinese isolates from different hosts. Additionally, the haplotypes from different hosts were the same in the populations of other countries.

**Fig. 2.**
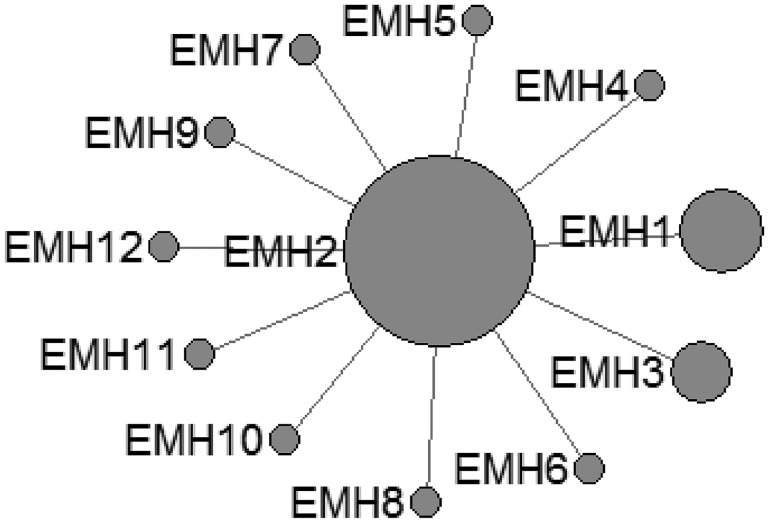
Haplotype network of the cytochrome c oxidase subunit 1 (*cox*1, 366 bp) gene of the *Echinococcus multilocularis* from the QTPA of China (EMH1) and other homologous sequences from GenBank constructed using NETWORK 5.0.

In *E. multilocularis*, 12 mtDNA haplotypes were found in all the isolates, which were also plotted a star-like network with one major haplotype (EMH2) (Fig. 2). The EMH1 haplotype came from three different hosts from two countries, and the EMH2 haplotype came from six different hosts from five countries, while the EMH4-EMH8 haplotypes all came from China but originated from different hosts.

**Fig. 3.**
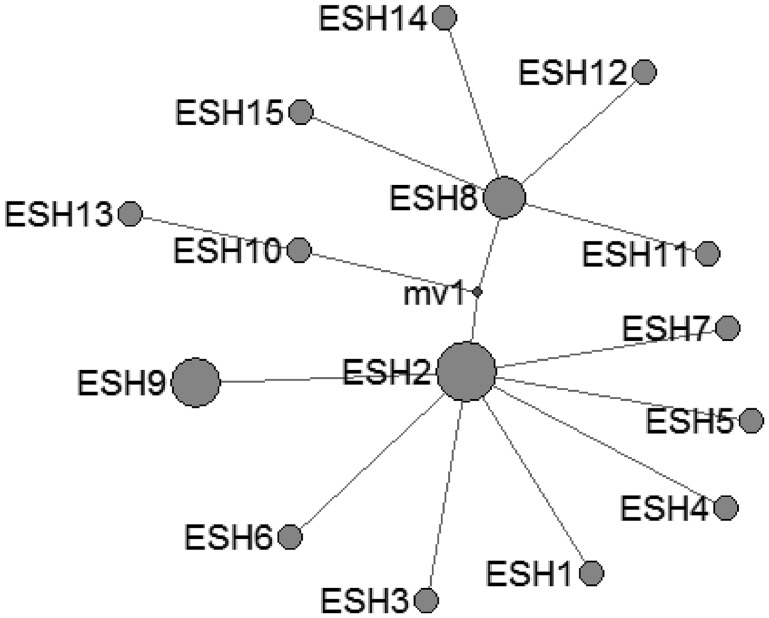
Haplotype network of the cytochrome c oxidase subunit 1 (*cox*1, 366 bp) gene of the *Echinococcus shiquicus* from the QTPA of China hosts (ESH1) and other homologous sequences from GenBank constructed using NETWORK 5.0.

In *E. shiquicus*, 15 mtDNA haplotypes were found in the all isolates from China originating from four different hosts. The network was complicated with one star-like (ESH1-7 an ESH9) configuration and two main sub-groups (ESH10-ESH13 and ESH8, ESH11-ESH12, ESH14-ESH15) (Fig. 3).

### Diversity and neutrality indexes

The diversity indexes for the China QTPA isolates of *E. granulosus* s.s. from three different host species are shown in Table 2. Haplotype diversity was high for all *E. granulosus* s. s. populations within these three host species and was highest in *E. granulosus* s. s. from humans and lowest in those from sheep. In contrast, nucleotide diversity was low for all host species and ranged from 0.006 to 0.008 because of the richness of single nucleotide substitutions.

**Table 2.** Diversity and neutrality indexes of the *Echinococcus granulosus* (sensu stricto) population calculated from the nucleotide sequence of the mitochondrial cytochrome c oxidase subunit 1 (*cox*1, 366 bp) gene. All *P* values for Tajima's *D* and Fu's *F*s were not significant (*P* > 0.05)

Host origin	*n*	*S*	*K*	*H*	Hd ± s.d.	*π* ± s.d.	Tajima's *D*	Fu's *F*s
Humans	93	21	2.74708	16	0.9028 ± 0.0140	0.007506 ± 0.004447	−0.97737	−3.47949
Yaks	91	21	2.60855	16	0.8781 ± 0.0161	0.007127 ± 0.004264	−1.08740	−3.89570
Sheep	38	15	2.35135	9	0.7909 ± 0.0416	0.006424 ± 0.003982	−1.09838	−1.06127
Total	222	21	2.619420	16	0.8731 ± 0.0109	0.007157 ± 0.004250	−0.67886	−1.84956

The neutrality indexes of *E. granulosus* s.s. populations from host species calculated with Tajima's *D* and Fu's *F*s tests are also shown in Table 2; the values for these two indexes were all negative, which indicates an excess of rare polymorphic sites and a significant deviation from neutrality.

### Pairwise fixation index values

The pairwise fixation indexes (Fst) for the *cox*1 sequences of populations of *E. granulosus* s.s. from different host species are shown in Supplementary Table 2. Low Fst values were observed for the majority of *E. granulosus* s. s. populations when compared in a pairwise manner with some populations showing negative values (humans/yaks). Since one common haplotype existed predominantly in the three host species, the Fst values between the populations were very small, ranging from 0.004 to 0.01. These low values implied that the populations were not genetically differentiated among these host species.

## Discussion

In this study, the genetic diversity and population structure of *Echinococcus* spp. in QTPA of China was investigated. Data were obtained *via* sequencing of the *cox*1 mitochondrial gene, which had historically been demonstrated to show intraspecific variability and had been used for the study of the population structure of *Echinococcus* spp. from other parts of the world (Bowles *et al*., 1992; Nakao *et al*., 2010; Casulli *et al*., 2012; Yanagida *et al*., 2012). Although the new data published in October, 2018, showed that *nad*5 gene (680bp) was a highly suitable marker used for the differentiation of *E. granulosus* s. s. genotypes (Kinkar *et al*., 2018*a*). Initially, we used the partial *cox*1 (366 bp) to distinguish three genotypes (G1–G3) described within *E. granulosus* s. s (Bowles *et al*., 1992). The results presented in this report indicated that G1 was the most dominant distinct species of *E. granulosus* s. s. in the hydatid cyst samples from humans and animals in regions of the QTPA, China. The epidemiological data of this present study were in line with some previous molecular studies from China that had demonstrated G1 was the most common and dominant species of *E. granulosus* s. s. in humans, livestock and wild animals in China (Ma *et al*., 2008, 2015; Yang *et al*., 2009; Liu *et al*., 2013; Yan *et al*., 2013; Zhong *et al*., 2014; Hu *et al*., 2015), Turkey (Erdogan *et al*., 2017), Iran (Farhadi *et al*., 2015; Arbabi *et al*., 2017), and Bulgaria (Marinova *et al*., 2017). Likewise, many epidemiological studies conducted in the majority of regions of the world have also indicated *E. granulosus* s. s. G1 as the predominant species (Laurimae *et al*., 2016; Roinioti *et al*., 2016; Avila *et al*., 2017; Debiaggi *et al*., 2017; Ehsan *et al*., 2017; Thapa *et al*., 2017).

The *cox*1 haplotypes of *E. granulosus* s. s. found in this study did not reveal apparent systematic phylogeographic structuring in the QTPA of China. The parsimony network analysis revealed that the haplotypes exhibited a star-like expansion from a main global founder haplotype, suggesting that the populations of the QTPA of China were not fully differentiated from each other. It was noteworthy that the founder was predominant in the world population, which suggested that one particular lineage of *E. granulosus* s. s. was globally widespread in geographically unrelated populations. Nakao suggested that the mutation of the founder haplotypes were not advantageous, because the amino acid sequences of the founder were the same as those of certain other haplotypes (Nakao *et al*., 2010). Recently, Kinkar *et al*. (2018*b*) had shown that there was no such a founder haplotype for geographically unrelated populations of *E. granulosus* s.s. G1 based on a significantly longer mtDNA sequences and using a much larger number of samples, Moreover, such a founder also cannot be identified in case of *E. granulosus* s.s. G3 (Kinkar *et al*., 2018*c*). The high haplotype diversity observed in *E. granulosus* s.s. coupled with the low nucleotide diversity observed in the isolates of the QTPA of China was similar to that reported from Tunisia (Boufana *et al*., 2014), Jordan, Iran (Yanagida *et al*., 2012) and Europe (Casulli *et al*., 2012). On the other hand, the population genetic structures of *E. granulosus* s. s. comparing among various endemic areas clarified its worldwide dispersal. The neutrality indexes of Tajima's *D* obtained in the current study were negative, which suggested a bias towards the presence of nucleotide variants and was a feature of recent population expansion. Additionally, the neutrality index values Fu's *F*s values was also negative for all populations, which indicated that the incidence of rare haplotypes was lower than expected under neutrality, and the values pointed to bottleneck events and/or purifying selection events that might have occurred in the past (Nakao *et al*., 2010; Boufana *et al*., 2014). Furthermore, the extremely low values of the fixation index Fst also supported genetic non-differentiation between the local populations, indicating limited gene flow.

The occurrence of most haplotypes (EgQH1, 3, 4, 7, 12, 13, 16) of *E. granulosus* s.s. in the QTPA of China was consistent with previously reported results (Ma *et al*., 2008, 2015). This distribution of the haplotypes indicates the importance of sheep and yak in maintaining potential reservoir infections for humans and definitive hosts, which further suggested it might cycle among these host species. The high frequency of the dominant *E. granulosus* s. s. haplotypes in the QTPA suggested that it may be the ancestral haplotype in the QTPA of China. In another surveillance report of *Echinococcus* isolates from the QTPA of China, a total of 105 haplotypes (H1-H105) were detected, and 177 variable sites were recorded in 521 samples using the *cox*1 mtDNA marker gene (Ma *et al*., 2015). Our results were different from these, our animal isolates collected from the slaughterhouse and the transported animals usually came from the same places, so the results were also different.

In the current study, *E. multilocularis* (*n* = 16) and *E. shiquicus* (*n* = 6) were present in pika isolates, and no co-infections were observed in individual isolated samples from the Golog Tibetan Autonomous Prefecture. Unfortunately, we were only allowed to conduct sampling in the Golog prefecture at this time, which provided an explanation for why only the same genotype was found here. In our future work, we will conduct sampling and analyses elsewhere, and more samples will be collected and analysed. The identified sequences were highly similar to the referenced *E. multilocularis* (AB033406) and *E. shiquicus* sequences (AB208064). *E. shiquicus* was first identified in the Tibetan fox *Vulpes ferrilata* (adult stage) and the plateau pika *Ochotona curzoniae* (larval stage) and was characterized based on its morphological, genetic and ecological features (Xiao *et al*., 2005, 2006). Thereafter, *E. shiquicus* infections in dogs were also reported in the eastern QTPA; *E. shiquicus* was being transferred from its natural host, the Tibetan fox to the domestic dog (Boufana *et al*., 2013), which will threaten human health, although no cases of *E. shiquicus* human infection have yet been reported. Fan *et al*., conducted a genetic diversity analysis of *E. shiquicus* isolates from the plateau pika in Darlag County of Qinghai Province, and the genetic diversity of the *nad*1 and *cox*1 genes was shown to vary by 0.1–1.2% and 0.1–1.0%, respectively, with 6 haplotypes ranging from 4.2 to 29.6% (Fan *et al*., 2016); however, only one *E. shiquicus* haplotype was found in this study. Fan *et al*. trapped 71 plateau pika samples; we obtained 22 samples and conducted one gene (*cox*1) sequence genetic diversity analysis. The number of samples and sample collection sites for analysis may need to be expanded in future studies. *E. multilocularis* represents the second greatest echinococcosis threat to the local people following *E. granulosus* s. l., and some *E. multilocularis* haplotypes are also shared by domestic animals (sheep, yaks, and dogs) and humans (Ma *et al*., 2015); however, only one *E. multilocularis* haplotype was found in the plateau pika and not in the yaks, sheep and humans in this study, but the threat of *E. multilocularis* to humans and livestock should not be neglected. *E. multilocularis* has been detected in domestic animals (dogs and cats) and wild hosts (deer mice, meadow voles and southern red backed voles), not only in China but also in other countries (Liccioli *et al*., 2014; Poulle *et al*., 2017). Echinococcosis can also be a foodborne disease, and host factors and environmental risk factors can also serve as important transmission routes that pose a threat to humans. Further studies are needed to cover many sample collection sites and a larger number of pathogen isolates, which may identify abundant strains and haplotypes in the hydatid cysts infecting human and animal populations of the QTPA, China.

## Supplementary material

For supplementary material accompanying this paper visit https://doi.org/10.1017/S0031182019000544. click here to view supplementary material
