# Supplementary material for: Genetic characterization of Echinococcus isolates from various intermediate hosts in the Qinghai-Tibetan Plateau Area, China
Source: Parasitology. 2019 Jun 21;146(10):1305–12. doi: 10.1017/S0031182019000544 (PMC6700708; doi:10.1017/S0031182019000544)
Supplement: Supplementary file 1 [file S0031182019000544sup.zip › S0031182019000544sup001.doc]

*Supplementary Table 1. All sequences from GenBank used for network of Echinococcus granulosus sensu stricto.*

| Accession NO. | Host | Country | Haplotype |
| --- | --- | --- | --- |
| MG548790 | Cattle | Turkey | EGH1 |
| KX685912 | Human | China | EGH1 |
| AB688612 | Ovis aries sheep | China | EGH1 |
| AB491449 | Ovis aries sheep | China | EGH1 |
| MH025946 | Human | Iran | EGH2 |
| MH050617 | Human | China | EGH2 |
| MH050615 | Human | China | EGH2 |
| MH050614 | Human | China | EGH2 |
| MH050612 | Human | China | EGH2 |
| MH050609 | Human | China | EGH2 |
| MH050608 | Human | China | EGH2 |
| MG792551 | livestock | Iran | EGH2 |
| MH397251 | Camel | Iran | EGH2 |
| MG548787 | Cattle | Turkey | EGH2 |
| MF544127 | Ovis aries | Turkey | EGH2 |
| MF544126 | Dog | Turkey | EGH2 |
| MG672260 | buffalo | India | EGH2 |
| MG672257 | Human | Kazakhstan | EGH2 |
| MG672256 | Goat | Iran | EGH2 |
| MG672254 | Human | Mongolia | EGH2 |
| MG672234 | Sheep | Iran | EGH2 |
| MG672237 | Sheep | Iran | EGH2 |
| MG672239 | Sheep | Iran | EGH2 |
| MG672243 | Sheep | Iran | EGH2 |
| MG672245 | Sheep | Iran | EGH2 |
| MG672246 | Cattle | Iran | EGH2 |
| MG672241 | Sheep | Iran | EGH2 |
| KX685899 | Human | China | EGH4 |
| MH050616 | Human | China | EGH5 |
| KX685893 | Human | China | EGH5 |
| AB491418 | Sheep | China | EGH5 |
| AY386209 | Sheep | China | EGH5 |
| AB688606 | Human | China | EGH5 |
| MG548791 | Cattle | Turkey | EGH6 |
| KX874725 | Human | Turkey | EGH6 |
| KY499563 | Human | Turkey | EGH6 |
| KX685894 | Human | China | EGH6 |
| KM513628 | Cattle | Iran | EGH6 |
| KM100577 | Sheep | Turkey | EGH6 |
| KM100576 | Sheep | Turkey | EGH6 |
| KM100574 | Sheep | Turkey | EGH6 |
| KJ540229 | Sheep | Iran | EGH6 |
| AB688595 | Sheep | Jordan | EGH6 |
| AB491430 | Human | China | EGH6 |
| DQ356880 | Human | China | EGH6 |
| MH050611 | Human | China | EGH7 |
| MG792552 | livestock | Iran | EGH7 |
| MG548788 | Cattle | Turkey | EGH7 |
| MG672255 | Human | Mongolia | EGH7 |
| MG672248 | Cattle | Iran | EGH7 |
| MG672242 | Sheep | Iran | EGH7 |
| MG672240 | Sheep | Iran | EGH7 |
| MG672238 | Sheep | Iran | EGH7 |
| MG672236 | Sheep | Iran | EGH7 |
| MG672232 | Sheep | Iran | EGH7 |
| MG672231 | Sheep | Iran | EGH7 |
| MG672205 | Sheep | Turkey | EGH7 |
| MG672203 | Sheep | Turkey | EGH7 |
| MG672201 | Sheep | Turkey | EGH7 |
| MG672200 | Sheep | Turkey | EGH7 |
| MG672198 | Sheep | Turkey | EGH7 |
| MG672197 | Sheep | Turkey | EGH7 |
| MG672192 | Sheep | Turkey | EGH7 |
| MG672188 | Cattle | Turkey | EGH7 |
| MG672184 | Cattle | Turkey | EGH7 |
| MG672182 | Cattle | Turkey | EGH7 |
| MG672181 | Cattle | Turkey | EGH7 |
| MH010310 | Human | Iran | EGH7 |
| MH010308 | Human | Iran | EGH7 |
| MG322623 | Sheep | Iran | EGH7 |
| MG322622 | Sheep | Iran | EGH7 |
| MG322619 | Sheep | Iran | EGH7 |
| MG322618 | Sheep | Iran | EGH7 |
| KX874726 | Human | Turkey | EGH7 |
| KX874724 | Human | Turkey | EGH7 |
| KX874723 | Human | Turkey | EGH7 |
| KX874722 | Human | Turkey | EGH7 |
| KX874720 | Human | Turkey | EGH7 |
| KX874718 | Human | Turkey | EGH7 |
| KX874717 | Human | Turkey | EGH7 |
| KX874713 | Human | Turkey | EGH7 |
| KX874712 | Human | Turkey | EGH7 |
| KX874711 | Human | Turkey | EGH7 |
| KY499576 | Human | Turkey | EGH7 |
| KY499575 | Human | Turkey | EGH7 |
| KY499574 | Human | Turkey | EGH7 |
| KY499573 | Human | Turkey | EGH7 |
| KY499572 | Human | Turkey | EGH7 |
| KY499571 | Human | Turkey | EGH7 |
| KY499570 | Human | Turkey | EGH7 |
| KY499569 | Human | Turkey | EGH7 |
| KY499567 | Human | Turkey | EGH7 |
| KY499566 | Human | Turkey | EGH7 |
| KY499565 | Human | Turkey | EGH7 |
| KY499564 | Human | Turkey | EGH7 |
| KY499561 | Human | Turkey | EGH7 |
| MH050611 | Human | China | EGH7 |
| KX685920 | Human | China | EGH7 |
| KX685891 | Human | China | EGH7 |
| MG792553 | Livestock | Iran | EGH10 |
| MG672244 | Goat | Iran | EGH10 |
| MF281539 | Sheep | China | EGH10 |
| JQ250807 | Goat | Iran | EGH10 |
| AB491448 | Yak | China | EGH10 |
| AB677809 | Sheep | China | EGH10 |
| EU178103 | Cattle | Turkey | EGH10 |
| DQ356882 | Dog | China | EGH10 |
| KX874707 | Human | Turkey | EGH13 |
| KP339047 | Dog | Iran | EGH13 |
| AB893242 | Human | Mongolia | EGH13 |
| AB688619 | Human | China | EGH14 |
| KX685906 | Human | China | EGH17 |
| KX685913 | Human | China | EGH18 |
| KX685895 | Human | China | EGH18 |
| AB688613 | Human | China | EGH18 |
| AB491415 | Sheep | China | EGH18 |
| MG280949 | Sheep | China | EGH20 |
| MG280948 | Sheep | China | EGH21 |
| MF281541 | Sheep | China | EGH23 |
| AB491423 | Human | China | EGH23 |
| DQ356879 | Human | China | EGH23 |
