# Supplementary material for: Genetic characterization of Echinococcus isolates from various intermediate hosts in the Qinghai-Tibetan Plateau Area, China
Source: Parasitology. 2019 Jun 21;146(10):1305–12. doi: 10.1017/S0031182019000544 (PMC6700708; doi:10.1017/S0031182019000544)
Supplement: Supplementary file 1 [file S0031182019000544sup.zip › S0031182019000544sup002.doc]

Supplementary Table 2. Pairwise fixation index (Fst) for *Echinococcus granulosus* (sensu stricto) populations from different hosts based on nucleotide data of the mitochondrial cytochrome c oxidase subunit 1 (*cox*1, 366 bp) gene.

|  | Humans | Yaks | Sheep |
| --- | --- | --- | --- |
| Humans | – |  |  |
| Yaks | -0.00774 | – |  |
| Sheep | 0.00940 | 0.00402 | – |
